# IMU-Based Virtual Road Profile Sensor for Vehicle Localization

**DOI:** 10.3390/s18103344

**Published:** 2018-10-07

**Authors:** Juhui Gim, Changsun Ahn

**Affiliations:** School of Mechanical Engineering, Pusan National University, Busan 46241, Korea; juhuigim@pusan.ac.kr

**Keywords:** road profile estimation, unknown input Kalman filter, virtual measurement, inertial sensor signals, localization

## Abstract

A road profile can be a good reference feature for vehicle localization when a Global Positioning System signal is unavailable. However, cost effective and compact devices measuring road profiles are not available for production vehicles. This paper presents a longitudinal road profile estimation method as a virtual sensor for vehicle localization without using bulky and expensive sensor systems. An inertial measurement unit installed in the vehicle provides filtered signals of the vehicle’s responses to the longitudinal road profile. A disturbance observer was designed to extract the characteristic features of the road profile from the signals measured by the inertial measurement unit. Design synthesis based on a Kalman filter was used for the observer design. A nonlinear damper is explicitly considered to improve the estimation accuracy. Virtual measurement signals are introduced for observability. The suggested methodology estimates the road profile that is sufficiently accurate for localization. Based on the estimated longitudinal road profile, we generated spectrogram plots as the features for localization. The localization is realized by matching the spectrogram plot with pre-indexed plots. The localization using the estimated road profile shows a few meters accuracy, suggesting a possible road profile estimation method as an alternative sensor for vehicle localization.

## 1. Introduction

Autonomous ground vehicles recognize the surrounding environment and autonomously generate driving commands. The possible benefits of autonomous vehicles in traffic are the reduction of traffic accidents, increased convenience for drivers, optimized traffic flow, and expeditious driving [1,2,3]. To enable autonomous driving, various techniques have been developed in the fields of advanced vehicle safety systems and intelligent transportation systems [4,5]. Among them, vehicle localization is one of the most important techniques for autonomous driving.

A typical method for vehicle localization involves using Global Positioning System (GPS) signals. GPS provides absolute position information with reliability within a few meters at a low cost. However, GPS requires a clear line of sight to satellites because GPS signals are vulnerable to visual obstacles and signal reflections [6,7]. Furthermore, the position accuracy of single-frequency GPS receivers is low (up to tens of meters) [8]. By using two-frequency GPS receivers, the accuracy can be improved to within meters, but the cost increases significantly [9,10,11,12].

GPS and other methods are fused to obtain accurate position information at an affordable price for production vehicles. For example, GPS is integrated with cameras [13], laser scanners [14], or inertial measurement units (IMUs) [15,16]. When GPS is available, additional sensors can be used to compensate for the GPS error [17,18], which is the main purpose of sensor fusion. If GPS is unavailable, the additional sensors are used as back-up sensors. Some back-up sensors can act as visual odometers, such as cameras and laser scanners, but they are vulnerable to weather and lighting conditions. When an IMU is used as a back-up sensor, dead reckoning is mainly used [19,20]. However, dead reckoning is only valid for a short time horizon because of error accumulation.

Nevertheless, an IMU has many advantages over other sensors. First, IMU signals are more robust against external interruptions than GPS and vision sensors because they are directly measured from the signal sources. In contrast, GPS or vision signals are measured through non-contact methods. Therefore, IMU signals are less affected by visual obstacles or weather conditions and well- reflect the response of the vehicle to excitations from the surrounding environment. Secondly, an IMU is cheap and readily mounted on most passenger vehicles for standard safety features such as electric stability control. Therefore, IMUs are available at a very low cost. If we can reduce the effect of error accumulation, an IMU can be an attractive back-up sensor for a localization system using GPS or a vision system when corresponding signals are interrupted or jammed. 

Because of these advantages, we focus on an alternative IMU-based localization method that is not based on dead reckoning. Our approach is based on the fact that the road surface at a position has characteristic longitudinal profiles that are distinct from those of other positions. A heuristic analogy is guessing one’s own location by feeling the vibrations and motions of a vehicle when on a familiar road. Some researchers have presented IMU-based localization methods that use pitch rate signals [21,22]. The shapes of measured pitch rate signals from a ground vehicle are compared with the shapes of pre-indexed pitch rate signals to identify the position. This method is based on different road profiles generating different vehicle motions. The method works without error accumulation, but the localization accuracy is affected by measurement noise because the pre-indexed signals and measured signals are compared in the time domain. Furthermore, vehicle dynamics signals, such as the acceleration and angular velocities, are dependent on the vehicle speed, which also affects the estimation accuracy.

The proposed approach is error-accumulation-free IMU-based localization that is less dependent on vehicle speed variations and robust to signal noise. The basic concept for our approach is comparing the profile of the current position with a pre-indexed road profile database, which does not require the addition of a GPS signal. This method may work as a redundant sensor in the localization process when in conjunction with a GPS or be a backup sensor when GPS is unavailable, which both increase the reliability of the navigation system. The suggesting localization method consists of two main parts. One is estimating the longitudinal road profile of the current location, and the other is feature matching between the estimated profile and the profiles in the database in the spectrogram framework. 

The dependency on the vehicle speed can be removed by using the estimated road profile, because the longitudinal road profile is time independent information. We estimate the road profiles with measured IMU signals because a cost-effective device for direct measurement of a road profile is unavailable. The estimation is based on a disturbance observer. However, estimating disturbance signals using only a body-attached IMU is impossible due to the lack of observability. Because of the observability issue, some researchers use additional sensors to estimate a longitudinal road profile without concerns on observability. Examples of such sensors are the suspension stroke sensor [23,24,25,26] and the velocity or acceleration sensors for the un-sprung mass [26,27,28], but this approach is not favored for production vehicles. Another approach found in the literature is that the sprung mass position is assumed to be the same as the road profile [29,30], but the assumption is not valid in general production vehicles. Our remedy is to introduce a virtual measurement that is based on a less stringent assumption than the assumption on the sprung mass position. The virtual measurement is an assumed value of the averaged road heights. The assumption is based on the hypothesis that the road surface variations in longer wavelengths than the vehicle wheelbase, such as the averaged road heights, are ignorable. Therefore, the virtual measurement is a low-pass-filtered road profile and set as 0. By introducing the virtual measurement, the observability conditions are satisfied without using additional sensors or a too stringent assumption.

The second part for localization is to match the road profiles between the estimated one and the pre-indexed one in the database. The immunity to noise is improved by a feature extraction method. We extract the features of a road profile in the frequency-distance domain. Characteristics are more clearly presented in the frequency domain than in the time domain, so we adopt a spectrogram technique for feature extraction [31]. A spectrogram is a two-dimensional plot in the time-frequency domain, so the characteristics of signals are displayed with richer information. Another benefit of a spectrogram is the easy rejection of measurement noise. In addition, by using the spectrogram, we can use image processing techniques for feature matching because the spectrogram is displayed in a two-dimensional domain.

The rest of the paper consists of the following. Section 2 presents the observer design for longitudinal road profile estimation. Section 3 presents the feature extraction and feature matching, and Section 4 shows the experimental validation. Section 5 presents the conclusions.

## 2. Observer Design for Road Profile Estimation

The pitch rate and vertical acceleration are the most representative signals obtained from road profiles. However, such signals are affected by many factors, such as the longitudinal acceleration, measurement noise, and vehicle velocity. Therefore, a localization method based on the measured pitch rate or vertical acceleration signals may not be robust. An input observer or a disturbance observer is used to achieve a signal that is only affected by the road profile. 

**Figure 1:**
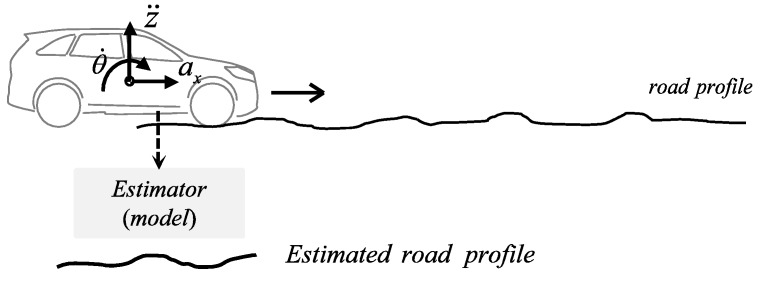
Concept of road profile estimation.

Figure 1 shows the concept of the road profile estimation. For straight driving, the main excitations of the vehicle’s dynamic responses are the vertical displacement of the tire-road contact points and the longitudinal acceleration. This information is used to estimate a road profile using measured IMU signals.

### 2.1. Vehicle Vertical Model

**Figure 2:**
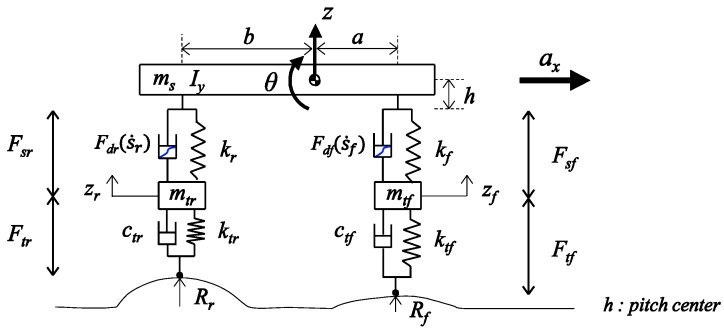
Half car model for vertical dynamics.

**Figure 3:**
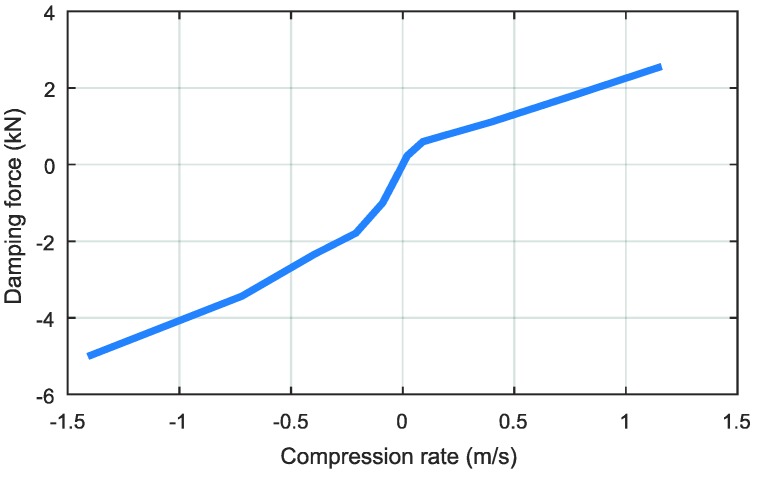
Damper model.

To capture the vertical and pitch dynamics, we consider a typical half car model, as shown in Figure 2. The tire and the suspension are modeled as spring-damper systems, but the damper of the suspension is modeled as a non-linear system. The nonlinearity of the suspension damper is one of the most significant factors in the accuracy of the estimation results. Therefore, the nonlinearity of the suspension dampers is explicitly considered in the model design. The damping force model is shown in Figure 3. 

The half car model with nonlinear dampers is expressed as a state space model:

(1) $$\begin{array}{l} \dot{x}=f(x)+B_{1}u+B_{2}d,\\ y=h(x), \end{array}$$

where *x* is a state vector, *u* is a vector of known inputs, and *d* is a vector of unknown inputs. The variables are defined as follows:

$$\begin{array}{l} x={\left[\begin{array}{cccccccc} s_{f} & {\dot{s}}_{f} & s_{r} & {\dot{s}}_{r} & z_{f} & {\dot{z}}_{f} & z_{r} & {\dot{z}}_{r} \end{array}\right]}^{T},\\ u=a_{x},\\ d={\left[\begin{array}{cccc} R_{f} & {\dot{R}}_{f} & R_{r} & {\dot{R}}_{r} \end{array}\right]}^{T},\\ y={\left[\begin{array}{cc} \dot{\theta } & \ddot{z} \end{array}\right]}^{T}, \end{array}$$

where *s_f_* and *s_r_* are the front and rear suspension strokes, respectively; and *R_f_*, *R_r_* and their time-derivatives are unknown inputs to the vehicle dynamics. These unknown inputs are determined by the road profiles and the vehicle velocity, and they cannot be easily measured using conventional sensors. *y* is a vector of the measurement signals and is a tuple of the pitch rate and vertical acceleration signals from the IMU sensor. 

### 2.2. Synthesis for Unknown Input Estimation

The states of a model can be estimated using conventional observers or Kalman filters. To estimate unknown inputs, Darouach proposed a modified Kalman filter for a linear system [32,33]. The summarized synthesis for a discrete linear system with unknown input *d_k_* is the following. In the following discrete system:

(2) $$\begin{array}{l} x_{k+1}=Fx_{k}+G_{1}u_{k}+G_{2}d_{k}+w_{k},\\ y_{k}=Hx_{k}+v_{k}, \end{array}$$

where *x_k_* is the state; *u_k_* is the known input; *y_k_* is the measurement; and *w_k_* and *v_k_* are zero mean Gaussian noise with covariance matrices of *W* and *V*, respectively. The state and unknown inputs are estimated from the modified Kalman filter:

(3) $$\begin{array}{l} {\bar{x}}_{k/k}=F{\hat{x}}_{k/k}+G_{1}u_{k},\\ {\hat{x}}_{k+1/k+1}={\bar{x}}_{k/k}+G_{2}{\hat{d}}_{k/k+1}+K_{k+1}^{x}\left\{y_{k+1}-H\left({\bar{x}}_{k/k}+G_{2}{\hat{d}}_{k/k+1}\right)\right\},\\ {\hat{d}}_{k/k+1}=K_{k+1}^{d}\left(y_{k+1}-H{\bar{x}}_{k/k}\right),\\ K_{k+1}^{x}={\left({\bar{P}}_{k/k}^{-1}+H^{T}V^{-1}H\right)}^{-1}H^{T}V^{-1},\\ K_{k+1}^{d}=P_{k+1/k+1}^{dx}H^{T}V^{-1}, \end{array}$$

where

$$\begin{array}{l} {\bar{P}}_{k/k}=F{\hat{x}}_{k/k}F^{T}+W,\\ P_{k+1/k+1}^{dx}=P_{k/k+1}^{d}G_{2}{}^{T}{\bar{P}}_{k/k}^{-1}{\left({\bar{P}}_{k/k}^{-1}+H^{T}V^{-1}H\right)}^{-1},\\ P_{k/k+1}^{d}={\left\{G_{2}{}^{T}H^{T}{\left(V+H{\bar{P}}_{k/k}H^{T}\right)}^{-1}HG_{2}\right\}}^{-1},\\ P_{k+1/k+1}^{x}={\left\{{\bar{P}}_{k/k}^{-1}+H^{T}V^{-1}H-{\bar{P}}_{k/k}^{-1}G_{2}{\left(G_{2}{}^{T}{\bar{P}}_{k/k}^{-1}G_{2}\right)}^{-1}G_{2}{}^{T}{\bar{P}}_{k/k}^{-1}\right\}}^{-1}. \end{array}$$

### 2.3. Linearization and Discretization of the Model

The synthesis can also be applied to a nonlinear system using the Jacobian matrices of the state transition and measurement functions. To design a disturbance observer, the nonlinear model (1) is linearized. At an operating point *x*_0_, the linearized model is:

(4) $$\begin{array}{l} \delta \dot{x}=A(t)\delta x+B_{1}\delta u+B_{2}\delta d,\\ \delta y=C(t)\delta x, \end{array}$$

where

$$\begin{array}{l} A(t)={\left[\frac{\partial f(x)}{\partial x}\right]}_{x_{0}},\;\;\;\;\;C(t)={\left[\frac{\partial h(x)}{\partial x}\right]}_{x_{0}},\\ B_{1}={\left[\begin{array}{cccccccc} 0 & \frac{a}{L}\frac{m_{s}}{I_{y}}h & 0 & -\frac{b}{L}\frac{m_{s}}{I_{y}}h & 0 & 0 & 0 & 0 \end{array}\right]}^{T},\\ B_{2}={\left[\begin{array}{cccccccc} 0 & -\frac{k_{tf}}{m_{tf}} & 0 & 0 & 0 & -\frac{k_{tf}}{m_{tf}} & 0 & 0\\ 0 & -\frac{c_{tf}}{m_{tf}} & 0 & 0 & 0 & -\frac{c_{tf}}{m_{tf}} & 0 & 0\\ 0 & 0 & 0 & -\frac{k_{tr}}{m_{tr}} & 0 & 0 & 0 & -\frac{k_{tr}}{m_{tr}}\\ 0 & 0 & 0 & -\frac{c_{tr}}{m_{tr}} & 0 & 0 & 0 & -\frac{c_{tr}}{m_{tr}} \end{array}\right]}^{T}. \end{array}$$

Finally, the linearized model (4) is discretized as a linear time-varying system for the observer design as follows:

(5) $$\begin{array}{l} x_{k+1}=F_{k}x_{k}+G_{1}u_{k}+G_{2}d_{k},\\ y_{k}=H_{k}x_{k}. \end{array}$$

### 2.4. Measurement Bias Model

The measurement signals include inevitable biases from the sensors or the installation error that usually occurs in a production vehicle. The measurement bias may cause accumulated errors in the estimation. If the sensor biases are not ignorable, the biases should be explicitly taken into account in the observer design. The biases are defined in the measurement vector *y_m_*:

(6) $$y_{m}=\left[\begin{array}{c} {\dot{\theta }}_{m}\\ {\ddot{z}}_{m} \end{array}\right]=\left[\begin{array}{c} \dot{\theta }+b_{\dot{\theta }}\\ \ddot{z}+b_{\ddot{z}} \end{array}\right],\;$$

where ${\dot{\theta }}_{m}$ is the measured pitch rate, $b_{\dot{\theta }}$ is the bias of the pitch rate sensor, ${\ddot{z}}_{m}$ is the measured vertical acceleration, and $b_{\ddot{z}}$ is the bias of the vertical acceleration sensor. The biases are usually constant or change slowly. Therefore, the bias model is assumed to be constant:

(7) $$\begin{array}{l} b_{\dot{\theta }}(k+1)=b_{\dot{\theta }}(k),\\ b_{\ddot{z}}(k+1)=b_{\ddot{z}}(k). \end{array}$$

### 2.5. Virtual Measurement for Observability

When the bias model is added to the linearized discrete system (5), the system model becomes larger and thus has 10 states, four unknown inputs, one known input, and two measurement signals. Before the observer design synthesis is applied to the system, the observability is investigated. The observability of an input observer can be tested using the following two conditions [34]:

(8) $$\begin{array}{l} 1.\;\;\;\;\;rank\left(O\right)=rank\left(\left[\begin{array}{c} {\underline{H}}_{k}\\ {\underline{H}}_{k}{\underline{F}}_{k}\\ \vdots \\ {\underline{H}}_{k}{\underline{F}}_{k}{}^{n-1} \end{array}\right]\right)=n,\\ 2.\;\;\;\;rank\left({\underline{H}}_{k+1}G_{d}\right)=rank(G_{d})=q, \end{array}$$

where *n* and *q* are the number of states and the number of unknown inputs, respectively. The states can be estimated if the observability matrix *O* has full column rank. The unknown inputs can be estimated if the matrices in the second condition have full column rank.

The second condition is not satisfied in our problem. A very easy resolution is to add additional measurement signals, but this requires additional cost. Therefore, we introduce a virtual measurement signal based on a reasonable assumption. The assumption is that the average road grade is zero in a distance horizon that is much longer than the vehicle wheel base. With this assumption, we introduce two virtual measurements, ${\bar{R}}_{f}$ and ${\bar{R}}_{r}$, which are the low-pass-filtered vertical displacements of the contact patches of the front and rear tires, respectively. The two virtual measurement signals are expressed as follows:

(9) $$\begin{array}{l} {\bar{R}}_{f}(k+1)=\alpha R_{f}(k)+(1-\alpha ){\bar{R}}_{f}(k),\\ {\bar{R}}_{r}(k+1)=\beta R_{r}(k)+(1-\beta ){\bar{R}}_{r}(k), \end{array}$$

where *α* and *β* are the filter parameters. The virtual measurements and *y_m_* are added to the final measurement vector:

(10) $$y_{aug}=\left[\begin{array}{c} y_{m}\\ {\bar{R}}_{f}\\ {\bar{R}}_{r} \end{array}\right]=\left[\begin{array}{c} y_{m}\\ 0\\ 0 \end{array}\right].\;$$

The final model for the road profile estimation is completed by adding the bias model and the virtual measurement model to the discretized vertical model (5):

(11) $$\begin{array}{l} x_{aug}(k+1)=F_{aug}(k)x_{aug}(k)+G_{1,aug}u(k)+G_{2,}{}_{aug}d(k),\\ y_{aug}(k)=H_{aug}(k)x_{aug}(k), \end{array}$$

where

$$\begin{array}{l} x_{aug}={\left[x^{T},\;\;\;\;b_{\dot{\theta }},\;\;\;\;b_{\ddot{z}},\;\;\;\;{\bar{R}}_{f},\;\;\;\;\;{\bar{R}}_{r}\right]}^{T},\\ y_{aug}={\left[\begin{array}{cccc} {\dot{\theta }}_{m} & {\ddot{z}}_{m} & {\bar{R}}_{f} & {\bar{R}}_{r} \end{array}\right]}^{T},\\ F_{aug}(k)=\left[\begin{array}{cc} F_{k} & 0_{10\times 4}\\ F_{21} & F_{22} \end{array}\right],\;F_{21}=\left[\begin{array}{cc} 0_{4\times 8} & \begin{array}{c} 0_{2\times 2}\\ \begin{array}{cc} \alpha & 0\\ 0 & \beta \end{array} \end{array} \end{array}\right],\;\;F_{22}=\left[\begin{array}{cc} \begin{array}{cc} 1 & 0\\ 0 & 1 \end{array} & 0_{2\times 2}\\ 0_{2\times 2} & \begin{array}{cc} 1-\alpha & 0\\ 0 & 1-\beta \end{array} \end{array}\right],\\ G_{1,aug}=\left[\begin{array}{c} G_{1}\\ 0_{4\times 1} \end{array}\right],\;\;G_{2,aug}=\left[\begin{array}{c} G_{2}\\ 0_{4\times 2} \end{array}\right],\;\;\\ H_{aug}=\left[\begin{array}{c} H_{k}\\ 0_{2\times 1} \end{array}\right]. \end{array}$$

This system satisfies the observability conditions, and the disturbance observer can be designed.

### 2.6. Road Profile Estimation Validation

**Figure 4:**
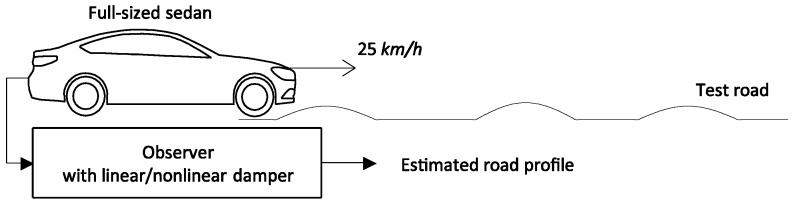
Simulation environment.

**Figure 5:**
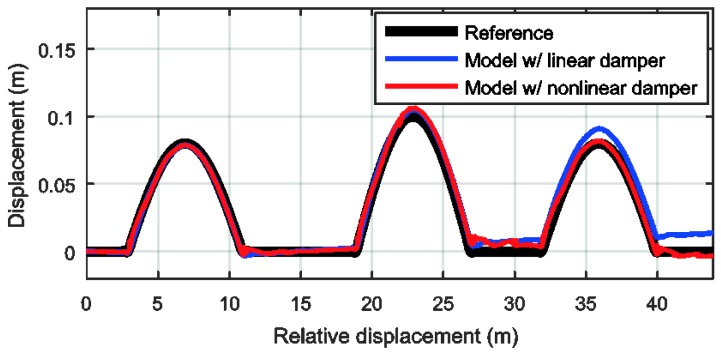
Estimated vertical displacement of front-tire contact patch.

We verified the estimation algorithm with a simulation and experiments. The simulation was performed with the virtual measurements vehicle dynamics simulation software Carsim^®^ (Mechanical Simulation Corporation, Ann Arbor, MI, USA). The vehicle was a full-sized sedan running at 25 km/h on a simple road profile, as shown in Figure 4. Figure 5 shows the road profile estimation results obtained using the system model with linear dampers and nonlinear dampers. The measurement signal does not have any noise or bias, so the error in the estimation mainly comes from the model error. Therefore, the result with nonlinear dampers shows a higher accuracy than the result with linear dampers.

**Figure 6:**
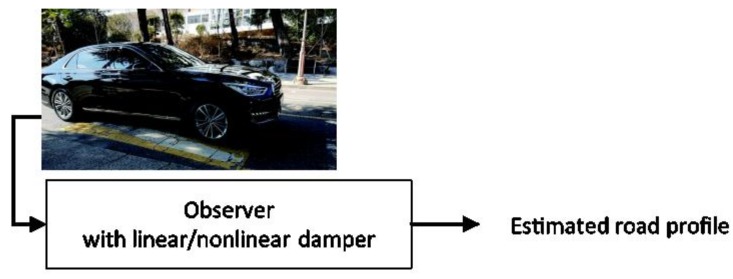
Experimental test of longitudinal road profile estimation.

**Figure 7:**
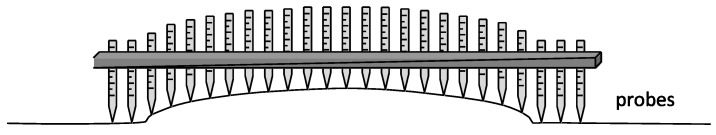
Road profile measurement device.

**Figure 8:**
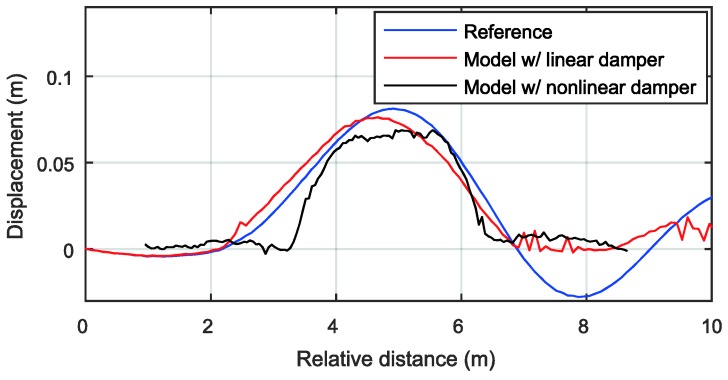
Estimated longitudinal road profile.

The experimental validation was performed using a speed bump, as shown in Figure 6. A full-sized sedan was driven on the speed bump, and the profile of the speed bump was measured using the ad-hoc device shown in Figure 7. The estimated results are plotted in Figure 8. The black line presents the measured profile. The observer with the nonlinear damper model provided more accurate estimates, especially after the vehicle ran over the bump. This occurred because a linear damper model would show a significant discrepancy from a true damper when the suspension stroke velocity is high. A high suspension stroke velocity usually occurs at sudden road profile changes such as speed bumps or pot holes.

## 3. Localization Using Estimated Road Profile

### 3.1. Feature Extraction

**Figure 9:**
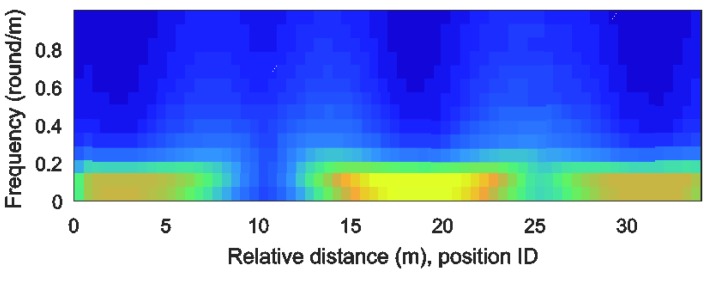
Spectrogram of the estimated road profile.

Unique and robust features are required to identify a specific location. The spectrogram shows the features of road profiles as an image in the time-frequency domain. By transforming a road profile to a spectrogram, we can apply various image feature extraction methods. Furthermore, we can extract robust features even when there is noise because the spectrogram has two feature dimensions: time and frequency. However, the vehicle speed affects the road profile distribution if the estimated road profiles are expressed in time. Therefore, we transform the road profile indexed in time into one that is indexed by the accumulated distance. By generating a spectrogram in the distance-frequency domain, the time dependency is removed. Figure 9 shows a spectrogram with the estimated road profile result from Figure 5. This spectrogram was generated with a moving window with dimensions of 10 m × 1 round/m. The color changes in the figure represent the road profile changes.

### 3.2. Feature Matching

**Figure 10:**
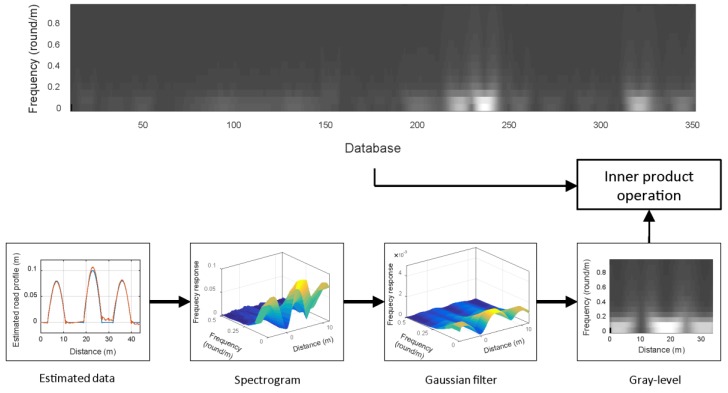
Normalized gray-level correlation process.

The feature matching involves determining the most corresponding index between a test sample and a portion of the database. We use an image comparison method based on normalized gray-level correlation. The database is a long image of a spectrogram that is obtained in advance. The test sample signal is transformed to a spectrogram and normalized to a gray level image. The similarity can be evaluated with the inner product operation:

(12) $$r=\frac{a\cdot b}{\left|a\right|\cdot \left|b\right|}=\frac{\sum_{i=1}^{M}\sum_{j=1}^{N}a(i,j)b(i,j)}{\sqrt{\sum_{i=1}^{M}\sum_{j=1}^{N}a{\left(i,j\right)}^{2}\sum_{i=1}^{M}\sum_{j=1}^{N}b{\left(i,j\right)}^{2}}},\;$$

where *a* is the normalized database vector, and *b* is the normalized test sample vector. If the correlation coefficient *r* has a high value near 1, the test sample image is similar to a portion of the database image. Among the *r* values obtained from scanning the database, the index of the highest value should be the most likely current location. Figure 10 shows the conceptual process of the feature extraction and matching.

## 4. Experimental Validation

**Figure 11:**
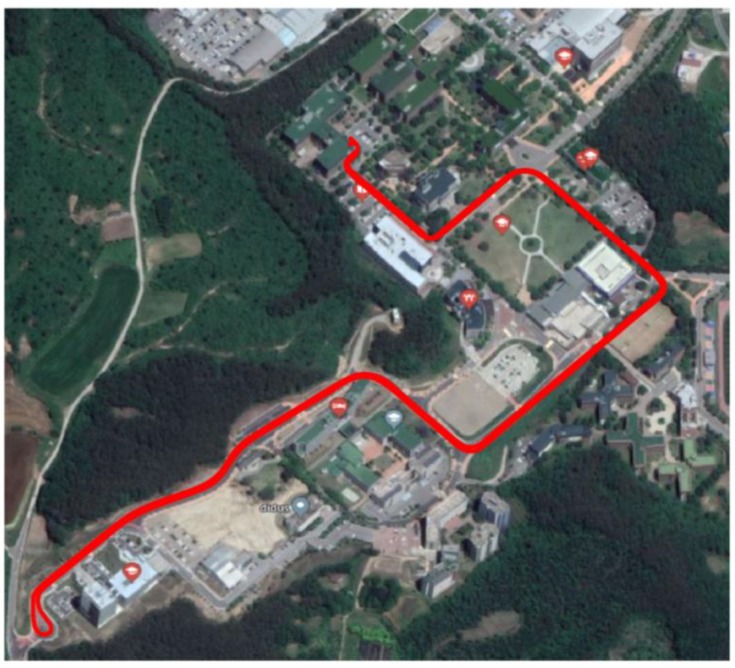
Test location for vehicle localization.

**Figure 12:**
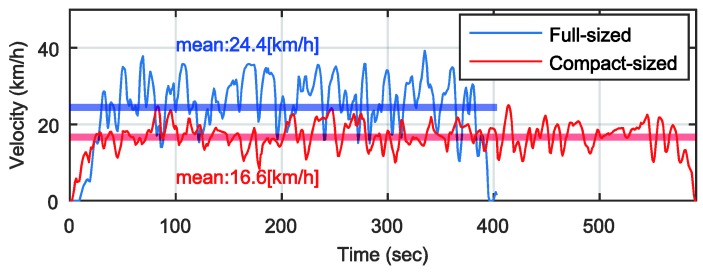
Vehicle velocities for localization tests.

**Figure 13:**
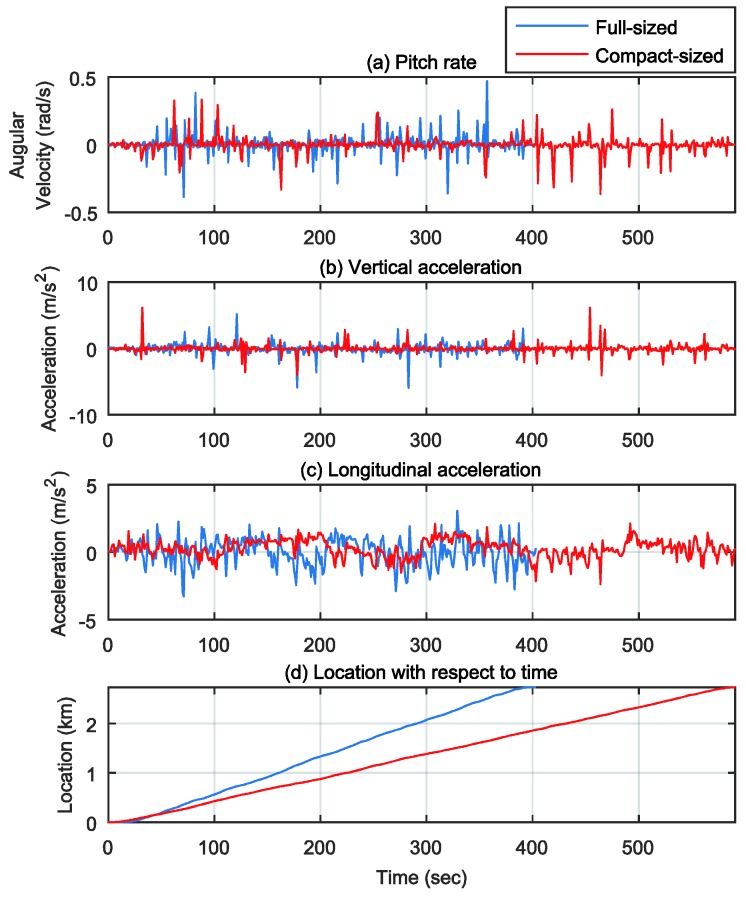
Measured signals from IMU sensor: (**a**) Pitch rate signals; (**b**) Vertical acceleration signals; (**c**) Longitudinal acceleration signals; (**d**) Location with respect to time.

To verify the localization method, we drove two different cars on the same road: a full-sized sedan and a compact sedan, as shown in Figure 11. The vehicle velocities are shown in Figure 12. The full-sized sedan ran at about 24.4 km/h, and the compact sedan ran at about 16.7 km/h. Figure 13a–c show the pitch rate, vertical acceleration, and longitudinal acceleration signals measured through the IMU for both cases, respectively. The signals in Figure 13a,b are the output *y*, and the signal in Figure 13c is the known input u in the estimator for the road profile. Figure 13d shows the driving distance with respect to time for both vehicles.

### 4.1. Consistency of Road Profile Estimatioin

**Figure 14:**
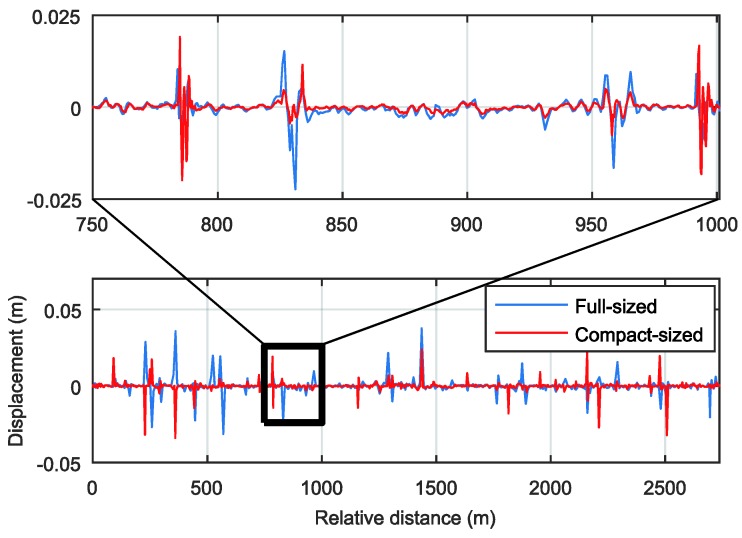
Estimated longitudinal road profile.

**Figure 15:**
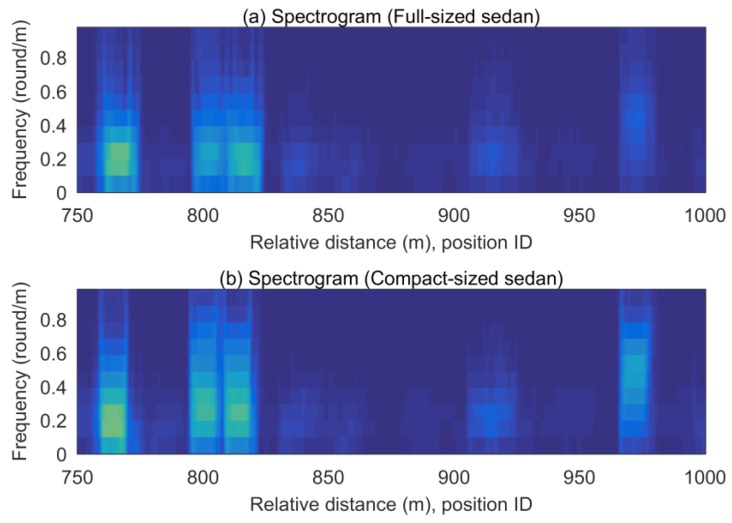
Spectrogram at 750–1000 m: (**a**) Full-sized sedan; (**b**) Compact-sized sedan.

The road profile should be extracted consistently for any type of vehicle at any speed. Figure 14 shows the estimated longitudinal road profile. The estimated profiles from the two vehicles show consistent patterns, even though the exact values are not identical. These consistent road profile results can provide the features for the localization in the same location, regardless of the speed or type of vehicle. The spectrograms of the estimated profiles from 750 to 1000 m in Figure 15 show more similar patterns. This occurs because the spectrogram is more affected by the distance and the frequency of the specific signal shapes in the distance domain than their magnitudes.

### 4.2. Localization

**Figure 16:**
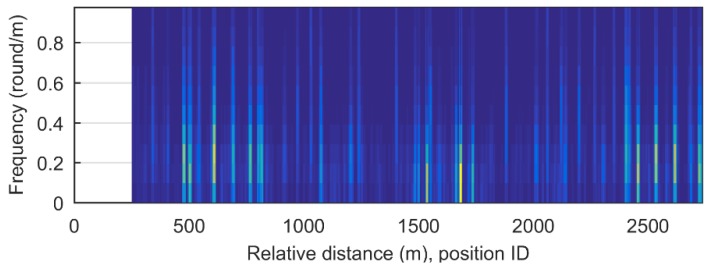
Reference spectrogram (pre-indexed database).

**Figure 17:**
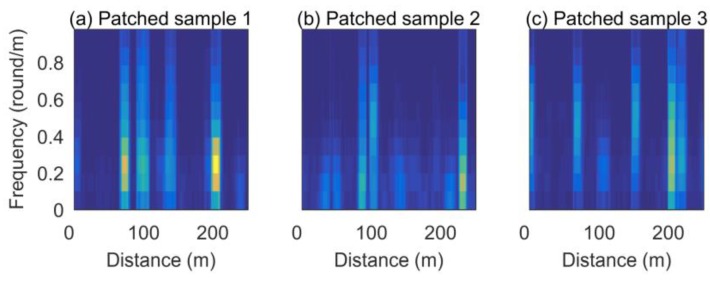
Spectrogram at three positions of test vehicles.

**Figure 18:**
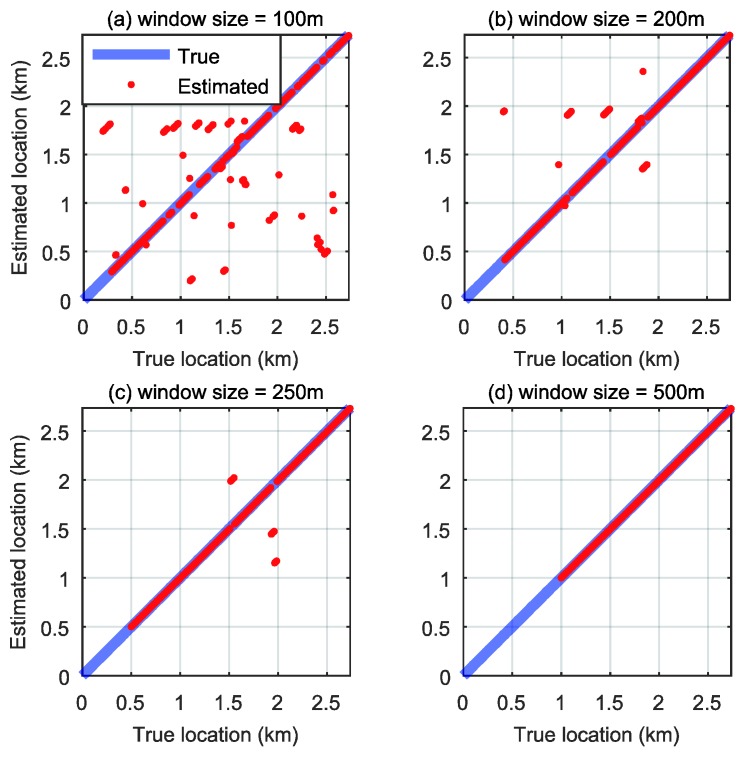
Localization result: (**a**) Window size = 100 m; (**b**) Window size = 200 m; (**c**) Window size = 250 m; (**d**) Window size = 500 m.

The estimated road profile from the full-sized sedan was used as a pre-indexed database, and that from the compact sedan was used as the test signal. Figure 16 shows the spectrogram transformed from the estimated road profile obtained with database signals with a window size of 250 m for the spectrogram generation. Figure 17 presents the spectrogram of the test runs obtained with this window. The localization was implemented by scanning the database spectrogram for the spectrogram of test signals. For example, for the spectrogram image obtained from the test vehicle shown in Figure 17a, the current position is about 400 m in the database index. In the same way, Figure 17b shows the image for 1450 m, and Figure 17c shows the image for 2200 m in the database index. The localization results for the total test signals are plotted in Figure 18.

Each graph in Figure 18 presents the localization result for when the lengths of the image moving window are 100, 200, 250, and 500 m. When the window size is larger, the estimated result is more accurate because the lager window includes more features. If the numbers and variety of features in the window are sufficient for localization, the localization accuracy increases, as shown in the case of the 500 m window. Notably, the localization results do not have accumulating error problems for any window sizes.

**Figure 19:**
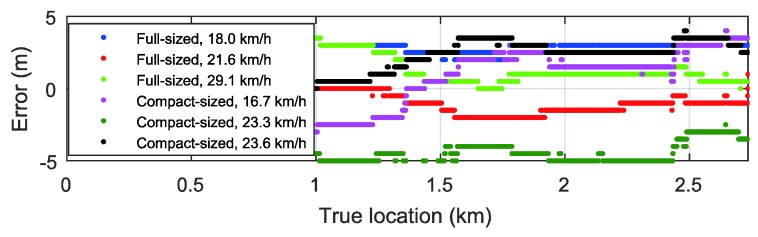
Estimation errors (window size = 500 m).

Figure 19 shows the error between the true position and the estimated positions with the measured signals from different environments when the window size is 500 m. The estimated localization results have under 5 m error without error accumulation.

Kuutti recently surveyed the state-of-the-art localization techniques [35]. He summarized that the localization techniques based on only one on-board sensor as a standalone offer a low accuracy and robustness for autonomous vehicles. The only GPS-based localization has under a 10 m accuracy [35], the only radar-based technique has an average accuracy of 10.5 m [36], and even the only camera-based localization has up to 20.5 m cumulative error [37]. The fusion techniques with vision sensors have under a meter accuracy; however, these techniques are vulnerable to illumination, the observation angle, the weather condition, and dynamic environments. The integrated GPS and IMU sensor localization technique has a relatively low accuracy of 7.2 m and error accumulation problem [38]. Another error-accumulation-free IMU-based localization method [21] has about a 5~10 m accuracy and estimation error deviations are large due to the signal noise. On the other hand, the suggested localization method has under 5 m error without an error accumulation problem using only an IMU sensor. Therefore, the suggested method can be applied as an alternative localization technique.

**Figure 20:**
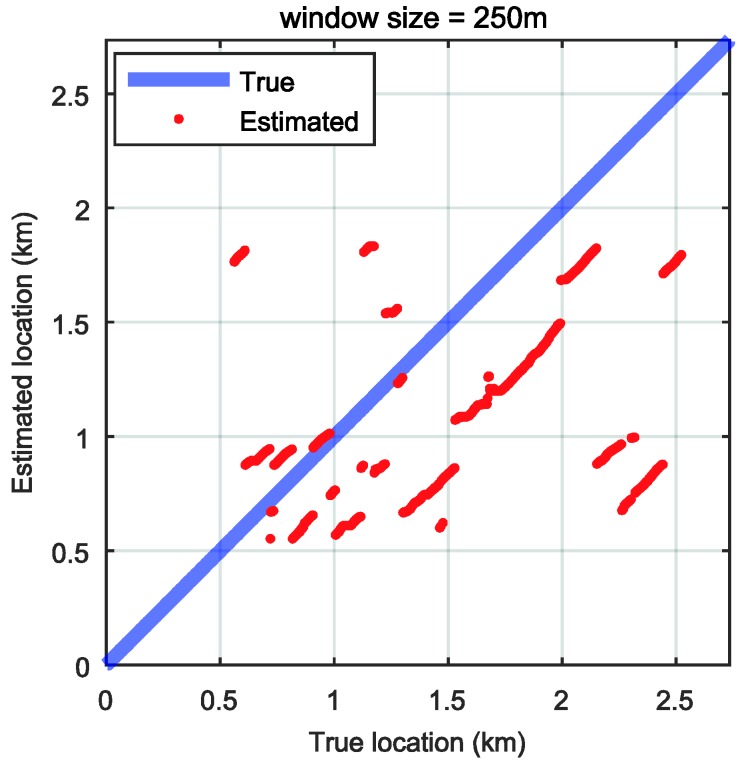
Localization result without road profile estimation (window size = 250 m).

We conducted the localization process with pitch rate signals to confirm the effect of the longitudinal road profile estimation in the localization process. The same methodology was used for localization, but the base signal is the pitch rate signal and not the road profile. Because the magnitude and frequency of the pitch rate signals are affected by the vehicle speed, the accuracy is much lower than the result with the estimated road profile, even with a larger window size, as shown in Figure 20.

## 5. Conclusions

This paper presented a non-dead-reckoning-based localization method using an IMU sensor without the concern of error accumulation. The localization concept matches the obtained signals with a chunk of the pre-indexed database. This method consists of two parts: (1) transforming the IMU signals in the time domain to the road profile in the distance domain through the disturbance observer; and (2) extracting and matching features in the spectrogram of the estimated road profile. The road profile estimation is implemented with the modified Kalman filter framework for unknown input estimation. The observer deals with the nonlinearity of the suspension for accurate estimation and introduces the virtual measurement to satisfy the observability condition without using additional sensors. The features for localization are in a distance-frequency spectrogram and the current position is identified using an image matching technique. The test results showed the validity of the suggested method with a few meters accuracy and repeatability without error accumulation, which are similar to or superior than those of only one-sensor based localization techniques without any sensor fusion. 

The suggested method has some advantages: (1) no error accumulation in the virtue of the time-independent road profile based localization; (2) a robust performance against vehicle speed variations; (3) richer features in a distance-frequency spectrogram than in the time-signal domain; (4) easy noise separation because of the road profiles transformed to the frequency domain; and (5) a higher possibility of implementation than other single sensor-based localization methods because of the low cost IMU. Therefore, the suggested method can be applied as a redundant sensor of GPS to improve the reliability of the navigation system or even applied as a back-up sensor when GPS is unavailable.
